# Standardizing the reporting of postoperative hypoparathyroidism following thyroidectomy: consensus statement from the European Society of Endocrine Surgeons, the American Association of Endocrine Surgeons, and the International Association of Endocrine Surgeons

**DOI:** 10.1093/bjs/znaf247

**Published:** 2025-11-13

**Authors:** Marcin Barczyński, Klaas Van Den Heede, James C Lee, Kerstin Lorenz, Radu Mihai, Olov Norlen, Kepal N Patel, Marco Raffaelli, Rebecca S Sippel, Tracy S Wang, Carmen C Solorzano

**Affiliations:** Department of Endocrine Surgery, Faculty of Medicine, Jagiellonian University Medical College, Kraków, Poland; Department of General and Endocrine Surgery, AZORG Hospital, Aalst, Belgium; Monash University Endocrine Surgery Unit, Alfred Hospital, Melbourne, Victoria, Australia; Department of Visceral, Vascular and Endocrine Surgery, Martin Luther University of Halle-Wittenberg, Halle (Saale), Germany; Department of Endocrine Surgery, Churchill Cancer Centre, Oxford University Hospitals NHS Foundation Trust, Oxford, UK; Department of Surgical Sciences, Uppsala University, Uppsala, Sweden; Department of Surgery, New York University Grossman School of Medicine and NYU Langone Health, New York, New York, USA; Division of Endocrine and Metabolic Surgery, Fondazione Policlinico Universitario Agostino Gemelli IRCCS, Rome, Italy; Centro di Ricerca in Chirurgia delle Ghiandole Endocrine e dell'Obesità, Università Cattolica del Sacro Cuore, Rome, Italy; Division of Endocrine Surgery, Department of Surgery, University of Wisconsin School of Medicine and Public Health, Madison, Wisconsin, USA; Department of Surgery, Section of Endocrine Surgery, Medical College of Wisconsin, Milwaukee, Wisconsin, USA; Department of Surgery, Division of Surgical Oncology and Endocrine Surgery, Vanderbilt University Medical Center, Nashville, Tennessee, USA

## Introduction

Postoperative hypoparathyroidism remains the most frequent complication following thyroid surgery, with reported incidence rates ranging from 14% to 60%^1–7^. While the majority of cases are transient and resolve within weeks, a substantial proportion (up to 25%) progress to persistent hypoparathyroidism, defined by sustained reductions in parathyroid hormone (PTH) and serum calcium levels beyond 6–12 months after surgery^1,2,4,5^.

Despite its prevalence and clinical impact, postoperative hypoparathyroidism continues to be variably defined across studies and institutions. Terminology such as ‘postoperative parathyroid failure’, ‘protracted hypoparathyroidism’, and ‘permanent hypoparathyroidism’ has been proposed to reflect the temporal evolution of the condition^6,7^. However, the lack of standardized definitions and reporting criteria has hindered meaningful comparisons across clinical studies and limited the development of consistent management strategies^7^.

Current approaches to hypoparathyroidism management range from short-term calcium and activated vitamin D supplementation to long-term replacement therapy and monitoring protocols^7^. These variations underscore the need for a unified framework to guide diagnosis, classification, and treatment.

In addition to harmonizing definitions, there is a critical need to identify and standardize core variables that should be reported in surgical research related to hypoparathyroidism. These include biochemical parameters (for example PTH and calcium levels), timing of measurements, patient symptoms, and therapeutic interventions. Consistent reporting of these variables is essential to improve data comparability, facilitate meta-analyses, and guide evidence-based practice.

Furthermore, hypoparathyroidism has emerged as a key quality marker in thyroid surgery. Its incidence and severity reflect not only surgical technique and intraoperative decision-making but also perioperative care and long-term follow-up. Establishing robust metrics for hypoparathyroidism, such as rates of transient *versus* permanent hypoparathyroidism, time to recovery, and need for chronic supplementation, can serve as benchmarks for surgical performance and institutional outcomes.

In response to this gap, the European Society of Endocrine Surgeons (ESES), the American Association of Endocrine Surgeons (AAES), and the International Association of Endocrine Surgeons (IAES) collaborated to develop a consensus statement using a structured Delphi methodology. This initiative aims to standardize the reporting of hypoparathyroidism in clinical research and publications (including definitions, core variables to be reported, and quality markers and metrics of thyroid surgery related to hypoparathyroidism) and provide a globally accepted reference for clinicians and researchers in endocrine surgery.

## Methods

### Composition of the working group, initial drafting, and development of consensus statements

In November 2023, the ESES launched a collaborative initiative aimed at establishing a consensus on how hypoparathyroidism following thyroid surgery should be reported in the scientific literature. As part of this effort, the ESES formally reached out to the leadership of the AAES and the IAES, proposing the formation of a joint taskforce to address this important and timely issue.

The proposal was met with unanimous support and all three societies appointed expert representatives in surgical thyroid disease to participate in the working group. Ultimately, the taskforce comprised 11 endocrine surgeons: 5 from the ESES (M.B., K.V.D.H., K.L., R.M., and M.R.), 3 from the AAES (K.N.P., R.S.S., and C.C.S.), and 3 from the IAES (J.C.L., O.N., and T.S.W.). This joint effort reflects a shared commitment across international societies to improve consistency and clarity in scientific reporting on hypoparathyroidism.

The working group was convened to conduct a comprehensive and current review of the literature, develop the initial draft, and formulate relevant consensus statements. This process was carried out through a series of web-based meetings, complemented by ongoing electronic correspondence to ensure continuity and collaboration.

A formal systematic review was deemed impractical and conceptually limiting for the purposes of this consensus statement. The primary challenge lay in the lack of a universally accepted definition of hypoparathyroidism and ‘surgical core variables’ relevant for prevalence of hypoparathyroidism that should be reported in scientific papers. As a result, it would have led to the exclusion of numerous pertinent studies. Conversely, many publications reference hypoparathyroidism without specifying the criteria applied, further complicating the selection process.

To address this, targeted literature searches were conducted by dedicated subgroups of three members assigned to each thematic section. The remaining members of the working group were invited to critically appraise the findings and contribute additional high-quality references where appropriate. In areas where published evidence was lacking, expert opinion served as the basis for several statements.

The initial draft and proposed statements were subsequently circulated among all working group members for review and refinement, before initiating the modified Delphi process.

### Panel composition and modified Delphi process

The modified Delphi method is a well-established process used to achieve consensus systematically and has recently been applied in several publications on thyroid cancer^8,9^. To reflect the diversity of clinical practice worldwide, while maintaining high expertise among contributing voters, the working group invited members of the ESES, the AAES, and the IAES, selected independently by each of the societies as experts in the field, to participate in a two-round modified Delphi process. The latter, along with the working group members, formed a panel of 92 panellists (full list in the Acknowledgements section) who voted on a dedicated invite-only electronic interface, powered by SurveyMonkey Inc. (San Mateo, CA, USA), thus providing coded data for analysis. Panellists voted on their level of agreement according to a Likert scale ranging from one (strongly disagree) to nine (strongly agree). The survey also allowed panellists to provide feedback and free-text comments on the statements during the first phase. Panellists with missing answers or with difficulties interpreting the statements were contacted individually through subsequent mailings for clarification. Appropriate revisions of the initial statements were made by the methodology leadership (M.B. and K.V.D.H.) supported by input from all taskforce experts according to the first survey feedback.

The statements were considered to meet consensus if there was a mean score of ≥7.0 (agree) and either ≤9 (10%) outlier responses or ≤14 (15%) outlier responses with <7 responses scoring <5 (disagreement). Near-consensus was assigned to statements with a mean of ≥6.50 and ≤18 (20%) negative outliers. Non-consensus was the default if the above conditions were not met. Outliers were defined by any value at least two Likert points away from the mean. Negative outliers refer to outliers where the response was that of disagreement (<5). The quality of evidence for each statement was stratified as high, moderate, low, or expert opinion, depending on the type of publication that data were extracted from. Data stemming from RCTs were considered to represent high-quality evidence, whereas those from non-randomized prospective trials were of moderate quality and data from retrospective analyses or case series were deemed to be of low quality. Where no supportive data were available, the term expert opinion was used.

### Finalization of position statement and organizational approval

The results of the survey were discussed in dedicated General Assembly (GA) meetings: during the 45th AAES annual meeting in Milwaukee, WI, USA on 19 May 2025, during the 11th ESES biennial conference in Izmir, Turkey on 24 May 2025, and by electronic vote for the IAES membership in May 2025 (as the IAES had no in-person meeting in 2025). For statements that reached near-consensus, a GA vote was undertaken to classify the statements as accepted or not. Voting options were limited to agree or disagree. An agreement level of >50% of total voters was set as the threshold for reaching consensus. The entire consensus statement document was finally approved by the GAs and thereby has full organizational support as a joint ESES/AAES/IAES consensus statement. A summary of the statements is presented in *Tables S1–S3*.

## Results

### Definition and incidence

Recently published meta-analyses have shown great inconsistency of definitions and diagnostic approaches to hypoparathyroidism after thyroid surgery^7^. The definitions of hypoparathyroidism included reduced PTH levels only, hypocalcaemia only, or a combination of both. In most cases, these groups could be subdivided into studies that included the presence or absence of symptoms to define the presence of hypoparathyroidism^7^. Moreover, in some patients, postoperative PTH levels, despite being within the normal range, are inadequate to maintain normal calcium levels and to avoid symptoms (relative parathyroid insufficiency)^10^.

Overall, this demonstrates significant variation in how hypoparathyroidism is defined across the studies, making direct and systematic comparisons of these parameters nearly impossible.

To address the challenges posed by complex and inconsistent diagnostic criteria, the latest statement from the American Thyroid Association (ATA) introduced a classification of hypoparathyroidism into three categories: biochemical hypoparathyroidism, clinical hypoparathyroidism, and parathyroid insufficiency (also referred to as relative hypoparathyroidism)^4^.

**Statement 1:** Biochemical postoperative hypoparathyroidism is defined by an undetectable or low PTH (less than the lower limit of the centre-specific reference range) with or without hypocalcaemia.

**Consensus:** Yes (8.088)

**Outliers:** 8

**Negative outliers:** 2

**Evidence level:** Moderate

A systematic review and meta-analysis recently analysed 188 studies on the definition and early diagnosis of hypoparathyroidism; it found that postoperative PTH measurements within 24 h had higher sensitivity and specificity than intraoperative PTH for predicting hypoparathyroidism, thresholds of <10 pg/ml and <15 pg/ml were both reliable for identifying patients at risk, no specific time point within the first 24 h (for example 1 h, 6 h *versus* 12 h *versus* 24 h) was superior, and any measurement within postoperative day 1 (POD1) was effective^7^. Nowadays, measuring PTH within 24 h is considered standard practice in many endocrine surgery centres. It allows for early identification of patients at risk of hypoparathyroidism, timely initiation of calcium and/or vitamin D supplementation, and safe discharge for individuals at low risk of hypoparathyroidism. This timing is also used in registry protocols and quality improvement initiatives to benchmark outcomes. In addition, measuring serum PTH levels 1–6 h after total or completion thyroidectomy provides high predictive accuracy for identifying the need for supplementation to prevent symptoms that may require readmission. This approach enables selective supplementation and supports safe early discharge, particularly in outpatient thyroidectomy protocols where timely discharge decisions are critical^8,9^.

**Statement 2:** The first postoperative PTH level should be measured within 24 h.

**Consensus:** Yes (8.022)

**Outliers:** 9

**Negative outliers:** 8

**Evidence level:** Moderate

**GA voting:** Agree: 78.25%, Disagree: 21.75%. In addition, 66.78% of voters preferred this measurement to be done 1–6 h after surgery.

Hypoparathyroidism is a clinically heterogeneous condition, where biochemical markers like PTH and calcium are essential for diagnosis, but symptom severity varies widely and may not align with laboratory values^11,12^.

**Statement 3:** Postoperative hypoparathyroidism may be an asymptomatic condition or it can become clinically apparent with a variety of manifestations ranging from mild numbness and tingling, muscle cramps, tetany, and seizures to life-threatening laryngospasm and cardiac arrhythmia.

**Consensus:** Yes (8.460)

**Outliers:** 3

**Negative outliers:** 0

**Evidence level:** Moderate

Several clinical and surgical factors have been proven to elevate the risk of hypoparathyroidism following thyroidectomy^12^:

- Severe obesity (BMI >40 kg/m^2^), as surgery is technically and metabolically more challenging.
- Vitamin D deficiency (25(OH)D3 ≤25 nmol/l), which predisposes patients to more prolonged and severe episodes of transient hypocalcaemia.
- Paediatric patients present unique challenges due to smaller parathyroid glands, prominent thymic tissue, and reactive lymph nodes, requiring meticulous surgical precision.
- Graves’ disease increases surgical complexity due to the thyroid’s hypervascular nature and the underlying inflammation.
- Thyroid cancer requiring central neck dissection may lead to inadvertent damage or devascularization of the parathyroid glands due to extensive tissue clearance.
- Combined thyroid and parathyroid procedures raise the risk of parathyroid suppression or accidental excision, especially when glands are small or difficult to identify.
- Reoperative thyroid surgery carries a higher risk due to scar tissue and altered anatomy.
- Parathyroid autotransplantation—whether into the neck or forearm—reflects intraoperative compromise and may signal increased risk of postoperative dysfunction.

Early substitution with oral calcium and active vitamin D should be routinely initiated in patients with biochemical hypoparathyroidism within 24 h post-surgery. This approach was shown to reduce the risk of symptomatic hypocalcaemia, including muscle cramps, paraesthesia, and cardiac irritability, and supported safe discharge planning. Use of standardized supplementation protocols should be helpful to prevent under-treatment and avoid emergency hospital readmissions^12,13^. Sessa *et al*.^14^ compared three protocols for preventing symptomatic hypocalcaemia after total thyroidectomy: PTH-driven selective postoperative supplementation with calcium and active vitamin D (calcitriol); high-dose routine preoperative and postoperative supplementation; and low-dose routine preoperative and postoperative supplementation. High-dose routine preoperative and postoperative supplementation resulted in the shortest hospital stays and the lowest rate of symptomatic hypocalcaemia. Although overall hypocalcaemia rates were similar across groups, PTH-driven selective supplementation alone had significantly more symptomatic cases. No readmission for hypoparathyroidism or hypercalcaemia occurred in the study. High-dose routine preoperative supplementation was deemed the most effective and safest approach, making it the protocol recommended by Sessa *et al*.^14^. In addition, maintaining appropriate serum magnesium levels is essential for PTH function. Low intracellular magnesium impairs PTH responsiveness due to its role as a cofactor for adenylate cyclase, while elevated serum magnesium may suppress PTH synthesis and secretion by activating the calcium-sensing receptor (CaSR)^12^.

**Statement 4:** Patients with biochemical hypoparathyroidism within 24 h after surgery should have oral calcium supplementation with or without an active vitamin D analogue started before discharge to reduce the risk of developing clinical manifestations of hypocalcaemia.

**Consensus:** Yes (8.311)

**Outliers:** 8

**Negative outliers:** 1

**Evidence level:** Moderate

**Statement 4A:** Serum calcium levels (albumin-adjusted and/or ionized) should also be measured.

**Consensus:** Yes (7.800)

**Outliers:** 11

**Negative outliers:** 7

**Evidence level:** Moderate

Adjustment protocols for calcium and vitamin D supplementation vary widely among external follow-up providers, leading to inconsistent management. To prevent iatrogenic suppression of PTH, excessive calcium intake, and potential renal complications, thyroid surgeons should proactively recommend a structured tapering strategy for supplementation^13^.

**Statement 5:** In patients with biochemical hypoparathyroidism and/or low serum calcium levels and/or manifestations of hypocalcaemia, measurements of calcium should be repeated as necessary to assure diagnosis and to allow tailoring of calcium and/or active vitamin D analogue supplements.

**Consensus:** Yes (8.188)

**Outliers:** 7

**Negative outliers:** 3

**Evidence level:** Moderate

According to the ATA statement, transient hypoparathyroidism refers to cases lasting <6 months following surgery, whereas permanent hypoparathyroidism persists beyond that time frame^4^. These two classifications are based on and expand upon existing literature, supported by extensive research and data. Relative hypoparathyroidism, though characterized by normal serum PTH and calcium levels, still necessitates treatment due to clinically relevant symptoms or signs of hypocalcaemia. While the ATA’s diagnostic criteria for hypoparathyroidism are valuable for identifying patients based on hypocalcaemia symptoms and guiding clinical care, they may not accurately reflect the true incidence—potentially leading to either underestimation or overestimation. Qiu *et al*.^5^ found that PTH levels recovered between 6 and 12 months after thyroid surgery with central neck dissection in 25.4% of patients diagnosed with permanent hypoparathyroidism at 6 months after surgery and that PTH levels recovered or did not recover after 12 months in 74.6% of patients. This phenomenon can be partly explained by the fact that most autotransplanted parathyroid glands typically require 2–6 weeks to gradually regain function, although, in some cases, full recovery may take several months^9^. Villarroya-Marquina *et al*.^11^ studied 854 patients undergoing total thyroidectomy and found that 14.5% of patients had postoperative hypoparathyroidism, with their parathyroid function recovering at different times (<6 months in 8.5%, 6–12 months in 2.5%, and >12 months in 1.4%; 4.2% of patients did not recover during follow-up). These data support the 12-month threshold for distinguishing transient from permanent hypoparathyroidism, as a substantial proportion of patients continue to show recovery of parathyroid function between 6 and 12 months after surgery. This approach is consistent with the most recent definition developed and approved by an international panel of experts presented as part of the best practice recommendations discussed at the Parathyroid Summit, held as a pre-Endocrine Society meeting in May 2024 (Boston, MA, USA), stating that a diagnosis of permanent hypoparathyroidism is confirmed ≥12 months after surgical intervention in the presence of hypocalcaemia (albumin-adjusted or ionized calcium) determined on two separate occasions (≥2 weeks apart) with inappropriately normal or low PTH levels^12^.

**Statement 6:** Temporary postoperative hypoparathyroidism is a condition that usually resolves within the first 6 months after surgery (but sometimes can last up to 12 months) with PTH and serum calcium levels within the reference range, without calcium and/or active vitamin D analogue supplements.

**Consensus:** Yes (7.928)

**Outliers:** 8

**Negative outliers:** 6

**Evidence level:** Moderate

**Statement 7:** Permanent postoperative hypoparathyroidism is a condition that continues for more than 12 months after surgery with persistently low serum calcium levels if not supplemented. The PTH level can be undetectable, low, or inappropriately low. To assure the diagnosis of this condition, there should be an attempt at stopping supplements within 12 months after surgery.

**Consensus:** Yes (7.722)

**Outliers:** 8

**Negative outliers:** 7

**Evidence level:** Moderate

### Surgical core variables to be reported

A meta-analysis of 188 studies on hypoparathyroidism after thyroid surgery revealed significant heterogeneity in the definitions and diagnostic criteria for hypoparathyroidism, highlighted the lack of consistency in the reporting of key surgical variables (such as number of parathyroid glands identified or preserved, use of autotransplantation, inadvertent excision confirmed by histology, and postoperative PTH and calcium levels), and recommended a unified framework for reporting to enable comparative analysis across studies and cohorts^7^.

A minimum data set of surgical core variables is essential to accurately define and classify hypoparathyroidism, benchmark surgical outcomes, and facilitate multicentre research and registry integration. This approach aligns with initiatives by the EUROCRINE Society (European Registry for Endocrine Surgery), the Collaborative Endocrine Surgery Quality Improvement Program (CESQIP), and the ATA, which advocate for structured data collection to improve quality and transparency with regard to thyroid surgery outcomes^4^.

**Statement 8:** There is a minimum set of surgical core variables that affect and define postoperative hypoparathyroidism following thyroid surgery that should be reported in research and publications. The aim of this approach is to improve the reporting in outcome-related publications allowing for comparisons between different studies and cohorts of patients.

**Consensus:** Yes (8.370)

**Outliers:** 2

**Negative outliers:** 0

**Evidence level:** Moderate

#### Risk factors

Postoperative hypoparathyroidism is multifactorial in origin, including compromised blood supply to the parathyroid glands, thermal injury from electrocautery, trauma such as haematoma or bruising, and inadvertent removal during surgery. These risks are significantly reduced if surgery is undertaken by high-volume thyroid surgeons, whose training and experience enables them to reliably differentiate the small parathyroid glands—typically weighing 30–50 mg and measuring <5 mm—from adjacent structures like adipose tissue, the thymus, lymph nodes, or thyroid tissue^12^.

On the other hand, patient-specific factors play a critical role in influencing the risk of hypoparathyroidism. These factors affect both the technical complexity of the procedure and the physiological resilience of the parathyroid glands.

A meta-analysis by Chen *et al*.^15^ identified a group of significant risk factors for postoperative hypocalcaemia that should be noted by the surgeon: hypoparathyroidism, OR 5.58; total thyroidectomy, OR 3.59; hypomagnesaemia, OR 2.85; preoperative vitamin D deficiency, OR 2.32; female sex, OR 1.49; thyroid malignancy, OR 1.85; thyroiditis, OR 1.48; substernal multinodular goitres, OR 1.70; parathyroidectomy, OR 1.58; central compartment neck dissection, OR 1.17; and modified radical neck dissection, OR 1.57.

#### Visual identification of the parathyroid glands

Visual identification of the parathyroid glands helps guide preservation, but does not guarantee function. Actually, published studies in this area show conflicting results. Riordan *et al*.^16^ showed that patients in whom a greater number of parathyroids had been identified had a significantly higher incidence of biochemical and symptomatic hypocalcaemia, and significantly lower postoperative PTH levels, than patients with fewer glands identified. Riordan *et al*.^16^ suggested that extensive dissection to identify all glands may increase the risk of manipulation and devascularization, leading to transient dysfunction. However, other studies and surgical protocols advocate for systematic identification to ensure preservation and reduce inadvertent excision—highlighting the lack of consensus^17^. Lorente-Poch *et al*.^18^ found that visualizing and preserving more parathyroid glands *in situ* during total thyroidectomy significantly reduces the risk of hypocalcaemia and permanent hypoparathyroidism. Patients with a lower PGRIS score (1–2 *versus* 3 or 4; where PGRIS stands for parathyroid glands remaining *in situ*) had higher rates of hypoparathyroidism.

#### Near-infrared autofluorescence (NIRAF) for identification of the parathyroid glands

Wang *et al*.^19^ presented in a meta-analysis of 24 studies involving 2062 patients and 6680 specimens that the diagnostic accuracy of NIRAF for identifying the parathyroid glands intraoperatively was high (sensitivity: 96%, specificity: 96%, area under curve: 0.99). Hence, NIRAF can be considered a highly accurate tool for systematic identification of the parathyroid glands, reducing the risk of inadvertent excision and hypoparathyroidism. This supports the broader principle that systematic identification—whether by visual inspection or adjunct technologies—is essential for preserving parathyroid function during thyroidectomy. It reinforces the need to include number of glands identified, preservation technique, and adjuncts used as core surgical variables in outcome reporting.

#### Inadvertently removed parathyroid glands identified at histology

In addition, the pathology report plays a crucial role in assessing the risk of hypoparathyroidism by providing objective confirmation of inadvertent parathyroid gland excision, which is a well-established risk factor for both transient and permanent hypoparathyroidism. Inadvertent parathyroidectomy during total thyroidectomy with central neck dissection for papillary thyroid carcinoma is common and involves the inferior glands more frequently in patients with extended resections and clinical N1a disease. Sitges-Serra *et al*.^20^ documented that transient and permanent hypoparathyroidism were more frequent after inadvertent parathyroidectomy (64% *versus* 46% and 15% *versus* 4%; *P* ≤ 0.03 each).

**Statement 9:** This minimum set of surgical core variables should include the following baseline parameters:

*Patient characteristics*

**Consensus:** Yes (8.136)

**Outliers:** 9

**Negative outliers:** 0

**Evidence level:** Moderate

*Disease characteristics*

**Consensus:** Yes (8.303)

**Outliers:** 4

**Negative outliers:** 0

**Evidence level:** Moderate

*Type and extent of thyroid surgery*

**Consensus:** Yes (8.617)

**Outliers:** 0

**Negative outliers:** 0

**Evidence level:** Moderate

*Intraoperative number of parathyroid glands identified and preservation technique(s) including intraoperative autotransplantation, and adjuncts used*

**Consensus:** Yes (7.966)

**Outliers:** 6

**Negative outliers:** 6

**Evidence level:** Moderate

*Postoperative laboratory findings*

**Consensus:** Yes (8.573)

**Outliers:** 1

**Negative outliers:** 1

**Evidence level:** Moderate

*Supplementation with calcium and/or vitamin D/active vitamin D analogue*

**Consensus:** Yes (8.471)

**Outliers:** 2

**Negative outliers:** 2

**Evidence level:** Low

*Final pathology report*

**Consensus:** Yes (7.775)

**Outliers:** 8

**Negative outliers:** 4

**Evidence level:** Moderate

*Length of follow-up*

**Consensus:** Yes (8.494)

**Outliers:** 2

**Negative outliers:** 1

**Evidence level:** Moderate

*Surgical volume*

**Consensus:** Yes (7.224)

**Outliers:** 14

**Negative outliers:** 5

**Evidence level:** Moderate

#### Bariatric surgery in anamnesis

A meta-analysis of 19 547 patients with a history of bariatric surgery has shown that they have a significantly greater risk of hypocalcaemia after thyroidectomy (30.6% *versus* 13.0%; OR 3.90; *P* = 0.005), with a heightened risk among those who have had a Roux-en-Y bypass procedure (38% *versus* 23%; OR 2.12; *P* = 0.020). Surgeons performing thyroid surgery should be aware of the increased risk of hypocalcaemia after thyroidectomy among these patients^21^.

#### Reoperative thyroid surgery

Reoperative thyroid surgery is a major risk factor for hypoparathyroidism due to increased technical difficulty and compromised parathyroid integrity. In a cohort of 2108 thyroid surgeries, reoperations were associated with a higher incidence of early hypocalcaemia, especially when fewer parathyroid glands were preserved *in situ*^22^. A multicentre study of 2631 patients found that completion thyroidectomy and central neck dissection significantly increased the risk of both transient and permanent hypoparathyroidism^23^.

**Statement 10:** Baseline patient characteristics as part of this minimum set of surgical core variables should include the following parameters:

*Sex*

**Consensus:** Yes (7.647)

**Outliers:** 10

**Negative outliers:** 3

**Evidence level:** Moderate

*Race and ethnicity*

**Consensus:** No (6.227)

**Outliers:** 24

**Negative outliers:** 13

**Evidence level:** Not Applicable

*BMI*

**Consensus:** No (6.025)

**Outliers:** 10

**Negative outliers:** 10

**Evidence level:** Not Applicable

*Preoperative serum calcium level*

**Consensus:** Yes (7.640)

**Outliers:** 11

**Negative outliers:** 4

**Evidence level:** Moderate

*Preoperative 25-OH vitamin D serum level*

**Consensus:** Yes (7.380)

**Outliers:** 9

**Negative outliers:** 2

**Evidence level:** Moderate

*Preoperative supplementation with calcium and/or vitamin D*

**Consensus:** Yes (7.303)

**Outliers:** 15

**Negative outliers:** 7

**Evidence level:** Low

**GA voting:** Agree: 59.72%, Disagree: 40.28%

*History of thyroid/parathyroid surgery*

**Consensus:** Yes (8.235)

**Outliers:** 8

**Negative outliers:** 2

**Evidence level:** Moderate

*History of bariatric surgery*

**Consensus:** Yes (7.707)

**Outliers:** 9

**Negative outliers:** 1

**Evidence level:** Low

*History of gastrointestinal malabsorption syndrome*

**Consensus:** Yes (7.730)

**Outliers:** 12

**Negative outliers:** 1

**Evidence level:** Low

**Statement 11:** Baseline disease characteristics as part of this minimum set of surgical core variables should include main diagnosis and indication for surgery.

**Consensus:** Yes (8.343)

**Outliers:** 4

**Negative outliers:** 0

**Evidence level:** Moderate

#### Surgical approach and extent of thyroid resection

To ensure consistency, transparency, and reproducibility in thyroid surgery research and clinical reporting, the procedure performed must be clearly specified. This includes both the technical approach and the extent of resection, as these factors significantly influence complication rates, recovery, and long-term outcomes.

The method used to access the thyroid gland should be explicitly stated. Common approaches include open/classical thyroidectomy, the transoral endoscopic thyroidectomy vestibular approach (TOETVA), robotic thyroidectomy, and other remote-access techniques^24,25^.

The amount of thyroid tissue removed must be clearly defined, as it directly affects the risk of complications such as hypoparathyroidism. Total or near total thyroidectomy, which involves complete removal of both lobes and the isthmus, is associated with the highest risk of hypoparathyroidism due to the proximity to the parathyroid glands^26^. Thyroid lobectomy (hemithyroidectomy) involves removal of one lobe ± the isthmus and the risk of hypoparathyroidism is extremely low. Lobectomy is increasingly the preferred approach for low-risk thyroid cancers^27^. Subtotal thyroidectomy involves partial preservation of thyroid tissue; it is rarely performed today, but may be used for specific benign conditions.

#### Central lymph node clearance

Central neck dissection involves removal of lymph nodes in the central compartment (level VI and sometimes VII) and is performed in conjunction with thyroidectomy for differentiated thyroid cancer. The extent (unilateral *versus* bilateral) and indication (prophylactic *versus* therapeutic) significantly influence the risk of postoperative hypoparathyroidism.

A prospective study showed lower postoperative PTH levels and higher rates of transient vocal cord palsy in bilateral central neck dissection compared with unilateral central neck dissection, though permanent complications were similar^28^.

Meta-analyses and randomized trials suggest that prophylactic central neck dissection for clinically node-negative thyroid cancer does not significantly reduce recurrence rates, but does increase transient hypoparathyroidism and nerve injury^29,30^.

#### Parathyroid reimplantation

Parathyroid autotransplantation is a widely used intraoperative strategy when the parathyroid glands are inadvertently devascularized or removed during thyroid surgery. While it aims to preserve parathyroid function, the number of glands reimplanted plays a critical role in determining postoperative outcomes, as multiple studies and meta-analyses have shown a positive correlation between the number of autotransplanted glands and the incidence of transient hypoparathyroidism^31–33^. One meta-analysis found that parathyroid autotransplantation was associated with a 1.75-fold increase in transient hypoparathyroidism, but no significant change in permanent hypoparathyroidism rates^32^. The most recent meta-analysis of 18 studies with >8000 patients confirmed these findings^31^. The key to preventing long-term complications is precise intraoperative identification and functional preservation of the parathyroid glands remaining *in situ*.

#### NIRAF and indocyanine green (ICG) angiography for identification and preservation of the parathyroid glands

Accurate intraoperative identification and preservation of the parathyroid glands is critical to preventing postoperative hypoparathyroidism. NIRAF technology leverages the intrinsic autofluorescence of parathyroid tissue under near-infrared light, allowing surgeons to visualize glands more reliably in real time. Multiple RCTs have demonstrated that NIRAF significantly increases the number of parathyroid glands identified during thyroidectomy^34^. In addition, NIRAF use has significantly reduced transient hypoparathyroidism due to better gland preservation^35,36^. However, there is no conclusive evidence yet that NIRAF reduces permanent hypoparathyroidism, though trends are favourable^37,38^.

ICG angiography is an emerging technique that allows real-time assessment of parathyroid gland perfusion during thyroidectomy. By injecting ICG dye and using near-infrared imaging, surgeons can visualize the vascular integrity of the parathyroid glands, which is a key predictor of postoperative function. Use of ICG angiography enables identification of well-perfused parathyroid glands, which are more likely to maintain function after surgery^39^. Vidal Fortuny *et al*.^40^ demonstrated that PTH levels on POD1 were normal in all patients who had at least one well vascularized parathyroid gland demonstrated during surgery using ICG angiography and none required treatment for hypoparathyroidism. In addition, Canali *et al*.^37^ showed that ICG angiography reliably predicted the vascularization of the parathyroid glands and obviated the need for postoperative measurement of calcium and PTH, and supplementation with calcium, in patients with at least one well perfused parathyroid gland.

This technique also facilitates selective autotransplantation of poorly perfused glands, potentially reducing the risk of permanent hypoparathyroidism^39,40^. In addition, this approach may improve decision-making during central neck dissection, where gland preservation is more challenging. This phenomenon is supported by data presented by Di Lorenzo *et al*.^41^, showing that the use of NIRAF imaging + ICG fluorescence decreased both transient and permanent hypoparathyroidism rates in patients undergoing total thyroidectomy and central neck lymph node dissection.

**Statement 12:** Surgical approach and extent of thyroid surgery should be clearly specified as part of this minimum set of surgical core variables and should include information on:

*Procedure performed (open/classical versus TOETVA versus robotic etc.; total thyroidectomy versus lobectomy versus subtotal/near-total resection)*

**Consensus:** Yes (8.393)

**Outliers:** 4

**Negative outliers:** 0

**Evidence level:** Moderate

*Central neck dissection (unilateral versus bilateral; prophylactic versus therapeutic)*

**Consensus:** Yes (8.629)

**Outliers:** 2

**Negative outliers:** 0

**Evidence level:** High

*Number of visualized parathyroids*

**Consensus:** Yes (7.629)

**Outliers:** 13

**Negative outliers:** 6

**Evidence level:** Moderate

*Number of parathyroids preserved in situ*

**Consensus:** Yes (7.741)

**Outliers:** 11

**Negative outliers:** 3

**Evidence level:** Moderate

*Number of parathyroids inadvertently removed and/or impossible to be preserved in situ*

**Consensus:** Yes (7.988)

**Outliers:** 4

**Negative outliers:** 3

**Evidence level:** Moderate

*Parathyroid autotransplantation (number of reimplanted glands)*

**Consensus:** Yes (8.426)

**Outliers:** 4

**Negative outliers:** 0

**Evidence level:** Moderate

*Use versus no use of NIRAF parathyroid detection systems (camera-based versus probe-based)*

**Consensus:** No (6.857)

**Outliers:** 10

**Negative outliers:** 10

**Evidence level:** High

**GA voting:** Agree: 66.32%, Disagree: 33.68%

*Use versus no use of intraoperative parathyroid angiography with ICG*

**Consensus**: No (6.380)

**Outliers:** 13

**Negative outliers:** 13

**Evidence level:** Not Applicable

*Use of energy-based devices for haemostasis*

**Consensus:** No (5.693)

**Outliers:** 48

**Negative outliers:** 27

**Evidence level:** Expert opinion

**Statement 13:** Short-term postoperative follow-up data necessary for assessing prevalence of early postoperative hypoparathyroidism should include serum PTH levels on the day of surgery or POD1 (based on surgeon/institutional protocol). In the case of low serum PTH or symptoms of hypocalcaemia, subsequent measurements of ionized or albumin-adjusted serum calcium are needed in the postoperative interval (days–weeks) to assess any need for or guide treatment of hypoparathyroidism.

**Consensus:** Yes (7.370)

**Outliers:** 10

**Negative outliers:** 9

**Evidence level:** Moderate

**GA voting:** Agree: 79.79%, Disagree: 20.21%

**Statement 14:** Short-term postoperative follow-up data should also include use of calcium supplementation (routine *versus* selective; oral only *versus* intravenous) and vitamin D or active vitamin D analogue prescription.

**Consensus:** Yes (8.370)

**Outliers:** 3

**Negative outliers:** 1

**Evidence level:** Moderate

#### Symptomatic hypoparathyroidism

Key elements for defining symptomatic hypoparathyroidism are: patient-reported symptoms, for example paraesthesia, muscle cramps, tetany, anxiety, and cognitive disturbances; and clinician-reported symptoms, for example Chvostek’s and Trousseau’s signs, seizures, and cardiac arrhythmias. It should be specified whether symptoms were collected via structured questionnaires, clinical interviews, or electronic health records. In addition, timing should be clarified (immediate postoperative interval *versus* long-term follow-up). Commonly accepted indicators of severity are: need for intravenous calcium—a direct marker of acute symptomatic hypocalcaemia; emergency department visits, which reflect uncontrolled symptoms or complications; and hospital readmissions, which indicate persistent or recurrent hypocalcaemia requiring inpatient care. These variables are essential not only for prevalence estimates but also for evaluating the burden of disease and effectiveness of interventions^14,42–44^.

**Statement 15:** To define prevalence of symptomatic hypoparathyroidism description of symptoms and how symptoms/signs were assessed and documented (patient-reported symptoms *versus* clinician-reported signs) should be specified. In particular, the need for intravenous calcium, visit(s) to the emergency department, and readmission(s) for hypoparathyroidism should be reported.

**Consensus:** Yes (8.179)

**Outliers:** 5

**Negative outliers:** 1

**Evidence level:** Moderate

**Statement 16:** Long-term follow-up data necessary for assessing prevalence of permanent hypoparathyroidism should include serum calcium (albumin-adjusted and/or ionized) and PTH levels at 12 months after surgery, and information if an attempt was undertaken at stopping supplements within 12 months after surgery with failure to stay off supplements due to low calcium and/or reoccurring symptoms.

**Consensus:** Yes (8.146)

**Outliers:** 8

**Negative outliers:** 4

**Evidence level:** Moderate

**Statement 17:** Pathology data should be reported with number (full/partial) of parathyroids identified in specimen.

**Consensus:** Yes (7.876)

**Outliers:** 6

**Negative outliers:** 3

**Evidence level:** Moderate

#### Baseline PTH serum level

Baseline PTH levels reflect the functional reserve of the parathyroid glands before surgery. Patients with a low preoperative PTH level may have subclinical parathyroid dysfunction or autoimmune thyroid disease (for example Hashimoto’s thyroiditis), making them more vulnerable to postoperative hypocalcaemia. Some studies suggest that preoperative PTH levels positively correlate with the postoperative PTH decline rate, suggesting a predictive value for transient hypoparathyroidism^10,44,45^.

#### Surgical volume

Surgeon experience directly influences the ability to identify and preserve the parathyroid glands and their vascular supply. Low-volume surgeons are more likely to inadvertently damage or remove parathyroid tissue^42^. Years of practice may reflect cumulative skill, but volume is a stronger predictor of outcomes in endocrine surgery. Adam *et al*.^46^ analysed data from 16 954 adults who underwent total thyroidectomy, identified through the Health Care Utilization Project–National Inpatient Sample, between 1998 and 2009. Of these patients, 47% had thyroid cancer and 53% had benign disease^46^. The likelihood of complications decreased as surgeon volume increased, with a clear threshold of improved outcomes at >25 total thyroidectomies per year^46^. Notably, 81% of patients were operated on by low-volume surgeons, who were associated with patients with higher complication rates and longer hospital stays^46^. The ATA also emphasizes that surgeon experience is a modifiable risk factor for hypoparathyroidism, recommending referral to high-volume centres for complex cases^4^. Thyroid cancer and autoimmune thyroid disease predict an increased risk of surgical morbidity and patients with these conditions should be operated on by high-volume surgeons. The oncological results of thyroid cancer surgery are significantly better when the surgery is performed by high-volume surgeons^47^.

**Statement 18:** In addition, preoperative serum PTH levels (low *versus* normal *versus* high) and surgical experience (low-volume: ≤25 thyroid procedures per year *versus* high-volume >50 thyroid procedures per year) along with years of practice might be helpful optional parameters describing landscape of risk factors for postoperative hypoparathyroidism.

**Consensus:** Yes (7.142)

**Outliers:** 14

**Negative outliers:** 6

**Evidence level:** Moderate

### Quality registries, markers, and metrics

In recent decades, there has been increasing use of multicentre or national databases that audit the workload and monitor outcome measures after endocrine surgery^48–52^. Decisions made at the inception of such registries impact the strength and extent of the data that can be used to answer a specific research question that is decided upon some years later. In general, it is considered that the information provided is more likely to represent the reality as the databases capture the practice of multiple surgeons, with a large spectrum of annual workload and expertise, and therefore such data might be more meaningful in comparison with the analysis provided by a research paper reporting outcome measures for cohorts of patients treated in highly specialized units.

The British Association of Endocrine and Thyroid Surgeons (BAETS) was one of the pioneers in this field when it established its initial online audit in the early 2000s. The National Endocrine Surgery Audit, known as the United Kingdom Registry of Endocrine and Thyroid Surgery (UKRETS), has collected data on thyroid, parathyroid, adrenal, and pancreatic endocrine procedures since 2005. The database now contains the outcomes and demographic data for >160 000 operations performed by >300 consultants across the UK. This is a large volume of cases with several million data points to guide surgical practices. The database tracks procedures involving the thyroid, parathyroid, and adrenal glands, as well as the pancreas, and is used to monitor outcomes, complications, and surgical workload. Several research papers have been written based on UKRETS data over the past 20 years. These papers and audit volumes have shown: how reoperation for dangerous bleeding in the neck has decreased with the use of haemostatic technology; how measuring intraoperative PTH in real time can help predict successful surgery and allow surgery to end; how the incidence of voice change can be reduced with nerve monitoring and the high incidence of recurrent laryngeal nerve palsy after thyroid/parathyroid surgery; and, lastly, that a higher surgical volume is associated with a shorter hospital stay and fewer complications after adrenal surgery (supporting centralization of surgery for adrenal cancer and bilateral tumours to higher-volume surgeons performing a minimum of 12 operations per year)^48,50,51^.

The Scandinavian Quality Register for Thyroid, Parathyroid and Adrenal Surgery (SQRTPA) was established in 2004 and since its inception it has received broad multidisciplinary support from the relevant professional associations. In 2009, the adrenal module was added^49^. The register is supported by the government-funded Council for National Quality Registers and the Swedish National Board of Health and Welfare. The register collects data from patients who have undergone surgical treatment for diseases of the thyroid, parathyroid, and adrenal glands. This means that the number of possible diagnoses for each type of procedure is extensive. It includes common diagnoses such as multinodular goitre and primary hyperparathyroidism, as well as rare conditions like parathyroid and adrenal cancer. The coverage rate and validity of the register are high, making it a robust tool for quality assurance and research. Numerous scientific publications focused on hypoparathyroidism have utilized data from SQRTPA over the past two decades^1,2,49,53,54^.

For the past decade, the EUROCRINE registry—launched in 2015 by the ESES—has become the largest repository, with >217 000 cases recorded to date^52^. EUROCRINE was initially funded as a project within the Health Programme of the European Union 2013. As of 2018, EUROCRINE is registered as a not-for-profit organization organized and duly registered under the laws of Austria for societies. The owner of the platform is Region Skåne, the County Council of Scania Region in Sweden. The EUROCRINE registry collects data to analyse diagnostic processes, indications for surgical treatment, types of surgical procedures, the use of resources, and outcomes. Data for quality control are analysed at the local hospital level and on an aggregate national and supranational level. Data are also used for clinical research and to identify and disseminate best clinical practice for the endocrine surgical procedures contributing to advancements in endocrine surgery and patient care. The EUROCRINE registry collects data on preoperative serum PTH levels (defined as low, normal, high, or not measured), use of parathyroid autotransplantation, number of parathyroid glands identified, use of autofluorescence to identify the parathyroid glands, use of calcium during admission (defined as preoperative medication for a reason other than hypoparathyroidism or routine protocol of the clinic irrespective of calcium/PTH values or treatment due to hypoparathyroidism), use of vitamin D on discharge from hospital, and postoperative PTH serum levels (low, normal, high, or not measured). No data have been published yet with regard to the incidence of permanent hypoparathyroidism, but individual surgeons/clinics contributing to the EUROCRINE database can see their own performance in comparison with national and international peers. This fosters transparency and encourages continuous improvement. The registry identifies effective treatment strategies and surgical techniques, helping clinicians refine their approach based on real-world evidence^55–59^. The ESES has recently launched an initiative to accredit surgical units across Europe as competence centres in endocrine surgery. To qualify, units must be registered in the EUROCRINE database, have at least one member who has passed the European Union of Medical Specialties (UEMS) Division of Endocrine Surgery exam, and be an active ESES member. This programme aims to uphold excellence in both surgical training and clinical practice^60,61^.

In the USA, the first national quality registry started in the Veterans Affairs (VA) hospital system in the early 1990s^62^. The VA quality improvement programme, called the National Surgical Quality Improvement Program (NSQIP), was so effective at improving surgical outcomes that the American College of Surgeons (ACS) launched a pilot programme in 2001 and, in 2004, established it as an ongoing quality registry for the ACS, available to all hospitals in the USA^63,64^. The NSQIP has been widely adopted across the USA and, while it was very effective at tracking and improving overall outcomes after surgical procedures, it lacked the procedure-specific complications that were felt to be so critical to the field of endocrine surgery^62–64^. In 2016, the NSQIP also recognized the need for procedure-specific data and created an optional module for patients undergoing thyroid surgery. As participation is optional, it captures only a subset of patients undergoing thyroid surgery each year. However, it has been approved as a Qualified Clinical Data Registry (QCDR) by the Centres for Medicare & Medicaid Services (CMS), allowing it to support US surgeons in meeting reporting requirements under the Merit-based Incentive Payment System (MIPS). Before development of this module, the AAES recognized the need for a quality registry that could track the outcomes that are specific to endocrine surgery and established the CESQIP in 2012^64,65^. This programme aims to improve the value of care delivered to patients. The CESQIP utilizes concepts of continuous quality improvement to improve outcomes and optimize costs. This is accomplished through patient-centred data collection, ongoing performance feedback to clinicians, and improvement based on analysis of collected data and collaborative learning. The programme is now utilized in >75 hospitals across the country and has collected quality data on >200 000 patients. The American Board of Surgery acknowledged the CESQIP as satisfying the requirements for an operative log and practice improvement registry for maintenance of certification. With time, the AAES recognized the need for a case-log programme to support the educational requirements and accreditation requisites for all AAES-accredited Comprehensive Endocrine Surgery Fellowship programmes. The Fellows’ module was launched in 2021 with the expectation that all Fellows-in-training will enter all endocrine procedures performed into the CESQIP. This allows the AAES Fellowship Accreditation Committee to monitor the progress of Fellows-in-training, ensure that the quality and breadth of training is similar across programmes, and monitor case volumes in specific areas (thyroid, parathyroid, adrenal, neuroendocrine tumours, ultrasonography etc.)^66^.

Very few national quality registries in Australia/Asia are available, although some large institutional databases report on the rate of hypoparathyroidism^67,68^.

**Statement 19:** Quality markers and metrics of thyroid surgery related to postoperative hypoparathyroidism should be reported in research and publications to allow for benchmarking analysis and quality improvement.

**Consensus:** Yes (7.840)

**Outliers:** 6

**Negative outliers:** 4

**Evidence level:** Moderate

**Statement 20:** The preferred way of reporting should be through the multicentre (for example NSQIP, EUROCRINE, CESQIP etc.) or national (for example SQRTPA, UKRETS etc.) databases that audit the workload and monitor outcome measures after thyroid surgery. However, the membership to these databases remains optional in the vast majority of healthcare environments.

**Consensus:** Yes (7.108)

**Outliers:** 17

**Negative outliers:** 7

**Evidence level:** Low

**GA voting:** Agree: 82.87%, Disagree: 17.13%

#### PTH as predictor of permanent hypoparathyroidism

Determining the frequency of long-term parathyroid dysfunction is challenging, as it typically requires a full year of follow-up and efforts to discontinue treatment—data that are often absent from many studies and registries. Recent research suggests that this condition may be more prevalent than previously assumed^2,53,54^. A faster method to estimate its incidence within a specific unit or hospital, without waiting for extended follow-up, would be highly valuable. It would enable clinicians to evaluate the quality of their patient care more effectively and facilitate research into strategies for preventing parathyroid dysfunction^1^.

This Swedish study analysed 1636 patients who underwent total thyroidectomy for benign conditions between 2005 and 2015, aiming to explore the relationship between early PTH levels and long-term parathyroid dysfunction^1^. Data were sourced from six hospitals and a national quality register, including clinical, surgical, and biochemical details. Findings revealed a notable rate of persistent parathyroid dysfunction, with some patients potentially receiving unnecessary prolonged treatment due to lack of reassessment. Importantly, patients with normal PTH levels within 24 h of surgery had a very low risk of developing permanent hypoparathyroidism. In contrast, 23% of patients showed low early PTH levels, which correlated with a 6.7% rate of long-term hypoparathyroidism^1^. The study suggests that early PTH measurement may serve as a useful predictor for permanent hypoparathyroidism, though further research is needed to confirm its reliability and refine treatment protocols.

**Statement 21:** Serum PTH level (when possible) should be obtained within 24 h after total or completion thyroidectomy as an obligatory surrogate biochemical marker of postoperative hypoparathyroidism. This parameter impacts management at the time of discharge. In addition, PTH level may be used as a quality marker allowing for estimation of the prevalence of early hypoparathyroidism and also to exclude a high prevalence of permanent postoperative hypoparathyroidism.

**Consensus:** Yes (7.590)

**Outliers:** 12

**Negative outliers:** 10

**Evidence level:** High

**GA voting:** Agree: 69.75%, Disagree: 30.25%

**Statement 21A:** Patients with normal PTH serum levels on POD1 have a very low risk of permanent hypoparathyroidism and are expected to need minimal monitoring and no calcium supplementation at discharge.

**Consensus:** Yes (7.863)

**Outliers:** 6

**Negative outliers:** 2

**Evidence level:** High

**Statement 21B:** Undetectable PTH serum level within 24 h after thyroid surgery may increase the risk of permanent parathyroid dysfunction and these patients require immediate onset of supplementation with calcium and an active vitamin D analogue and further close follow-up.

**Consensus:** Yes (7.965)

**Outliers:** 6

**Negative outliers:** 4

**Evidence level:** Moderate

**Statement 22:** Some preoperative parameters, intraoperative events, and postoperative follow-up data are obligatory to be reported in publications, as they are considered quality metrics of thyroid surgery that allow for more comprehensive risk assessment of the permanent hypoparathyroidism state.

**Consensus:** Yes (7.704)

**Outliers:** 9

**Negative outliers:** 2

**Evidence level:** Moderate

**Statement 22A:** Some preoperative parameters like 25-OH vitamin D serum level and PTH serum level should be reported when possible and available.

**Consensus:** Yes (7.879)

**Outliers:** 3

**Negative outliers:** 2

**Evidence level:** Moderate

**Statement 22B:** Some intraoperative events like number of parathyroid glands identified visually with confidence/preserved *in situ*/reimplanted should be reported.

**Consensus:** Yes (7.613)

**Outliers:** 7

**Negative outliers:** 5

**Evidence level:** Moderate

**Statement 22C:** Some postoperative follow-up data including inadvertent excision of parathyroid gland(s) confirmed by histology, PTH serum level within 24 h of the operation, need for intravenous calcium, visit(s) to the emergency department, and readmission(s) for hypoparathyroidism, and use of calcium/vitamin D supplements during early (6 weeks), and long-term (12 months) follow-up should be reported.

**Consensus:** Yes (8.045)

**Outliers:** 7

**Negative outliers:** 2

**Evidence level:** Moderate

#### Estimating permanent hypoparathyroidism: limits of prescription data

Tracking transient *versus* permanent hypoparathyroidism requires extended monitoring and multiple biochemical data points (for example PTH and calcium levels), which are often missing or inconsistently recorded in quality registries. Using prescription records for active vitamin D analogues over 12 months offers a practical workaround, especially in large-scale or retrospective studies where clinical data are incomplete. However, prescription data may not accurately reflect clinical status. Some patients may continue supplementation unnecessarily, while others with permanent hypoparathyroidism may be missed due to non-adherence or lack of documentation. Drug registry data cannot capture symptom severity, biochemical trends, or physician rationale for treatment continuation or cessation. Hence, while secondary sources like drug registries can offer a rough estimate of permanent hypoparathyroidism prevalence, they should be interpreted cautiously and ideally supplemented with clinical data to ensure accuracy. This approach is pragmatic, but not definitive.

**Statement 23:** The number of data points and the length of follow-up needed in quality registries to make a perfect calculation of transient and true permanent hypoparathyroidism rate after thyroid surgery may be difficult to reach. Hence, alternatively active vitamin D analogue use or prescription for over 12 months after thyroid surgery collected from a secondary source such as an insurance claims registry or a national prescribed drug register may be a feasible approximation, although it probably underestimates or overestimates the true prevalence of permanent hypoparathyroidism somewhat.

**Consensus:** No (6.722)

**Outliers:** 10

**Negative outliers:** 10

**Evidence level:** Low

**GA voting:** Agree: 72.32%, Disagree: 27.68%

These statements reinforce the urgent need for standardized, detailed, and transparent reporting practices in thyroid surgery to improve patient outcomes and enable meaningful comparisons across institutions. While 23 consensus statements were agreed upon in total, particular ones focused on quality markers and metrics (statements 19–23) emphasize the importance of early biochemical monitoring, structured data collection, and the limitations of indirect prevalence estimation methods. Together, they lay the groundwork for a unified global approach to managing and evaluating postoperative hypoparathyroidism.

## Final approval in the dedicated GA meetings

After a thorough discussion of the results, a separate vote was held to approve the above position statement in its entirety during the GA of the AAES, and ESES Annual Meetings, and by the GA of the IAES by an electronic vote. Of the 297 participants, 276 (92.93%) voted in favour of the consensus statement and 21 (7.07%) voted against the consensus statement.

This consensus statement, formally approved by members of the ESES, the AAES, and the IAES, provides a unified framework for the definition of hypoparathyroidism and reporting of surgical core variables, quality markers, and metrics related to the risk of hypoparathyroidism. It is intended to guide future research and publications on surgical outcomes in patients undergoing total or completion thyroidectomy, enabling greater consistency, comparability, and quality benchmarking across institutions and studies.

## Supplementary Material

znaf247_Supplementary_Data

## Data Availability

Data inquiry can be made available upon reasonable request to the corresponding author.
