# Supplementary material for: Standardizing the reporting of postoperative hypoparathyroidism following thyroidectomy: consensus statement from the European Society of Endocrine Surgeons, the American Association of Endocrine Surgeons, and the International Association of Endocrine Surgeons
Source: Br J Surg. 2025 Nov 13;112(11):znaf247. doi: 10.1093/bjs/znaf247 (PMC12612623; doi:10.1093/bjs/znaf247)
Supplement: znaf247_Supplementary_Data [file znaf247_supplementary_data.docx]

Supplementary Table S1: Summary of statements concerning definition, surgical core variables, and quality markers/metrics needed to be reported in surgical research and publications concerning postoperative hypoparathyroidism following total or completion thyroidectomy.

| **Statement No** | **Statement text** | **Mean score**  **(n=92 experts)** | **Outliers**  **No** | **Negative outliers**  **No** | **Status after Delphi process part 1^a^** | **Status after Delphi process part 2^b^** | **GA vote^b^**  **Agree -Disagree**  **(n=297 experts, including 88 ESES, 111 AAES, and 98 IAES)** | **Quality of evidence** |
| --- | --- | --- | --- | --- | --- | --- | --- | --- |
| 1 | Biochemical postoperative hypoparathyroidism is defined by an undetectable or low PTH (less than the lower limit of the centre-specific reference range) with or without hypocalcemia. | 8.088 | 8 | 2 | Consensus | N/A | NA | Moderate |
| 2 | The first postoperative PTH level should be  measured within24 h. | 8.022 | 9 | 8 | Near-consensus | N/A | Agree: 78.25%, Disagree:21.75% | Moderate |
|  | The first postoperative PTH level should be measured within **24 hours (preferably 1 to 6 hours).** | 7.702 | 12 | 10 |  | Near-consensus | Agree: 66.78%, Disagree: 33.22% | Moderate |
| 3 | Postoperative hypoparathyroidism may be an asymptomatic condition, or it can become clinically apparent with a variety of manifestations ranging from mild numbness and tingling, muscle cramps, tetany, seizures to life-threatening laryngospasm and cardiac arrhythmia. | 8.460 | 3 | 0 | Consensus | N/A | N/A | Moderate |
| 4 | Patients with biochemical hypoparathyroidism within 24 hours after surgery should have oral calcium supplementation with or without active vitamin D analogue started prior to discharge to reduce the risk of developing clinical manifestations of hypocalcemia. | 8.311 | 8 | 1 | Consensus | N/A | N/A | Moderate |
| 4A | Serum calcium levels (albumin-adjusted and/or ionized) should also be measured. | 7.8 | 11 | 7 | Consensus | N/A | N/A | Moderate |
| 5 | Patients with biochemical hypoparathyroidism and/or low serum calcium levels and/or manifestations of hypocalcemia, measurements of calcium should be repeated as necessary to assure diagnosis and to allow tailoring of calcium and/or active vitamin D analogue supplements. | 8.188 | 7 | 3 | Consensus | N/A | N/A | Moderate |
| 6 | Temporary postoperative hypoparathyroidism is a condition that resolves within the first 12 months postoperatively with PTH and serum calcium levels within the reference range, without calcium and/or active vitamin D analogue supplements. | 7.588 | 11 | 10 | Near-consensus | N/A | N/A | Moderate |
|  | Temporary postoperative hypoparathyroidism is a condition that **usually** resolves within the first **6 months** postoperatively **(but sometimes can last up to 12 months)** with PTH and serum calcium levels within the reference range, without calcium and/or active vitamin D analogue supplements. | 7.928 | 8 | 6 | N/A | Consensus | N/A | Moderate |
| 7 | Permanent postoperative hypoparathyroidism is a condition that continues for more than 12 months postoperatively with persistently low serum calcium levels if not supplemented. The PTH level can be undetectable, low or inappropriately low. To assure the diagnosis of this condition, there should be an attempt at stopping supplements within 12 months postoperatively. | 7.722 | 8 | 7 | Consensus | N/A | N/A | Moderate |

^a^ For near consensus approved/rejected by the general assembly vote

^b^ GA: General assembly vote, applicable only for statements with near-consensus

N/A: not applicable

Supplementary Table S2: Summary of statements concerning surgical core variables to be reported in surgical research and publications concerning postoperative hypoparathyroidism following total or completion thyroidectomy.

| **Statement No** | **Statement text** | **Mean score**  **(n=92 experts)** | **Outliers**  **No** | **Negative outliers**  **No** | **Status after Delphi process part 1^a^** | **Status after Delphi process part 2^b^** | **GA vote^b^**  **Agree -Disagree**  **(n=297 experts, including 88 ESES, 111 AAES, and 98 IAES)** | **Quality of evidence** |
| --- | --- | --- | --- | --- | --- | --- | --- | --- |
| 8 | There is a minimum set of surgical core variables that affect and define postoperative hypoparathyroidism following thyroid surgery which should be reported in research and publications. The aim of this approach is to improve the reporting in outcome-related publications allowing for comparisons between different studies and cohorts of patients. | 8.37 | 2 | 0 | Consensus | N/A | N/A | Moderate |
| 9 | This minimum set of surgical core variables should include the following baseline parameters: **PATIENTS CHARACTERISTICS** | 8.136 | 9 | 0 | Consensus | N/A | N/A | Moderate |
| 9 | This minimum set of surgical core variables should include the following baseline parameters: **DISEASE CHARACTERISTICS** | 8.303 | 4 | 0 | Consensus | N/A | N/A | Moderate |
| 9 | This minimum set of surgical core variables should include the following baseline parameters: **TYPE AND EXTENT OF SURGERY** | 8.617 | 0 | 0 | Consensus | N/A | N/A | Moderate |
| 9 | This minimum set of surgical core variables should include the following baseline parameters: **Intraoperative number of parathyroid glands identified and preservation technique(s) including intraoperative autotransplantation, and adjuncts used** | 7.966 | 6 | 2 | Consensus | N/A | N/A | Moderate |
| 9 | This minimum set of surgical core variables should include the following baseline parameters: **Postoperative laboratory findings** | 8.573 | 1 | 1 | Consensus | N/A | N/A | Moderate |
| 9 | This minimum set of surgical core variables should include the following baseline parameters: **Supplementation with calcium and/or vitamin D/active vitamin D analogue** | 8.471 | 2 | 2 | Consensus | N/A | N/A | Moderate |
| 9 | This minimum set of surgical core variables should include the following baseline parameters: **Final pathology report** | 7.775 | 8 | 4 | Consensus | N/A | N/A | Moderate |
| 9 | This minimum set of surgical core variables should include the following baseline parameters: **Length of follow-up** | 8.494 | 2 | 1 | Consensus | N/A | N/A | Moderate |
| 9 | This minimum set of surgical core variables should include the following baseline parameters: **Surgeon(s) volume** | 7.224 | 14 | 5 | Consensus | N/A | N/A | Moderate |
| 10 | Baseline patient’s characteristics as part of this minimum set of surgical core variables should include the following parameters: **Age** | 7.820 | 7 | 2 | Consensus | N/A | N/A | Moderate |
| 10 | Baseline patient’s characteristics as part of this minimum set of surgical core variables should include the following parameters: **Sex** | 7.647 | 10 | 3 | Consensus | N/A | N/A | Moderate |
| 10 | Baseline patient’s characteristics as part of this minimum set of surgical core variables should include the following parameters: **Race and ethnicity** | 6.227 | 24 | 13 | No consensus | N/A | N/A | N/A |
| 10 | Baseline patient’s characteristics as part of this minimum set of surgical core variables should include the following parameters: **Body mass index (BMI)** | 6.772 | 24 | 7 | Near-consensus | N/A | N/A | N/A |
|  | Baseline patient’s characteristics as part of this minimum set of surgical core variables should include the following parameters: **Body mass index (BMI)** | 6.025 | 10 | 10 | N/A | No consensus | N/A | N/A |
| 10 | Baseline patient’s characteristics as part of this minimum set of surgical core variables should include the following parameters: **Preoperative serum calcium level** | 7.640 | 11 | 4 | Consensus | N/A | N/A | Moderate |
| 10 | Baseline patient’s characteristics as part of this minimum set of surgical core variables should include the following parameters: **Preoperative 25-OH vitamin D serum level** | 7.438 | 9 | 2 | Consensus | N/A | N/A | Moderate |
| 10 | Baseline patient’s characteristics as part of this minimum set of surgical core variables should include the following parameters: **Preoperative supplementation with calcium and/or vitamin D** | 7.303 | 15 | 7 | Near-consensus | N/A | Agree: 59.72%, Disagree: 40.28% | Low |
|  | Baseline patient’s characteristics as part of this minimum set of surgical core variables should include the following parameters: **Standardized preoperative supplementation with calcium and/or vitamin D (if done)** | 6.807 | 10 | 10 | N/A | Near-consensus | Agree: 58.25%, Disagree: 41.75% | Low |
| 10 | Baseline patient’s characteristics as part of this minimum set of surgical core variables should include the following parameters: **History of thyroid/parathyroid surgery** | 8.235 | 8 | 2 | Consensus | N/A | N/A | Moderate |
| 10 | Baseline patient’s characteristics as part of this minimum set of surgical core variables should include the following parameters: **History of bariatric surgery** | 7.707 | 9 | 1 | Consensus | N/A | N/A | Low |
| 10 | Baseline patient’s characteristics as part of this minimum set of surgical core variables should include the following parameters: **History of gastrointestinal malabsorption syndrome** | 7.730 | 12 | 1 | Consensus | N/A | N/A | Low |
| 11 | Baseline disease characteristics as part of this minimum set of surgical core variables should include main diagnosis and indication for surgery. | 8.348 | 4 | 0 | Consensus | N/A | N/A | Moderate |
| 12 | Surgical approach and extent of thyroid surgery should be clearly specified as part of this minimum set of surgical core variables and should include information on: **Procedure performed (open/classical vs TOETVA vs robotic etc.; total thyroidectomy vs lobectomy vs subtotal/near-total resection)** | 8.393 | 4 | 0 | Consensus | N/A | N/A | Moderate |
| 12 | Surgical approach and extent of thyroid surgery should be clearly specified as part of this minimum set of surgical core variables and should include information on: **Central neck dissection (unilateral vs. bilateral; prophylactic vs. therapeutic)** | 8.629 | 2 | 0 | Consensus | N/A | N/A | High |
| 12 | Surgical approach and extent of thyroid surgery should be clearly specified as part of this minimum set of surgical core variables and should include information on: **Number of visualized parathyroids** | 7.629 | 13 | 6 | Consensus | N/A | N/A | Moderate |
| 12 | Surgical approach and extent of thyroid surgery should be clearly specified as part of this minimum set of surgical core variables and should include information on: **Number of parathyroids preserved in situ** | 7.741 | 11 | 3 | Consensus | N/A | N/A | Moderate |
| 12 | Surgical approach and extent of thyroid surgery should be clearly specified as part of this minimum set of surgical core variables and should include information on: **Number of parathyroids inadvertently removed and/or impossible to be preserved in situ** | 7.988 | 4 | 3 | Consensus | N/A | N/A | Moderate |
| 12 | Surgical approach and extent of thyroid surgery should be clearly specified as part of this minimum set of surgical core variables and should include information on: **Parathyroid autotransplantation (number of reimplanted glands)** | 8.426 | 4 | 0 | Consensus | N/A | N/A | Moderate |
| 12 | Surgical approach and extent of thyroid surgery should be clearly specified as part of this minimum set of surgical core variables and should include information on: **Use of near-infrared autofluorescence (NIRAF) parathyroid detection systems (camera-based vs. probe-based)** | 7.067 | 20 | 6 | Near-consensus | N/A | Agree: 43.16%, Disagree: 56.84% | N/A |
|  | Surgical approach and extent of thyroid surgery should be clearly specified as part of this minimum set of surgical core variables and should include information on: **Use versus no use of near-infrared autofluorescence (NIRAF) parathyroid detection systems (camera-based vs. probe-based)** | 6.857 | 10 | 10 | N/A | Near-consensus | Agree: 66.32%, Disagree: 33.68% | High |
| 12 | Surgical approach and extent of thyroid surgery should be clearly specified as part of this minimum set of surgical core variables and should include information on: **Use of intraoperative parathyroid angiography with indocyanine green (ICG).** | 6.910 | 27 | 8 | Near-consensus | N/A | N/A | N/A |
|  | Surgical approach and extent of thyroid surgery should be clearly specified as part of this minimum set of surgical core variables and should include information on: **Use versus no use of intraoperative parathyroid angiography with indocyanine green (ICG)** | 6.380 | 13 | 13 | N/A | No consensus | N/A | N/A |
| 12 | Surgical approach and extent of thyroid surgery should be clearly specified as part of this minimum set of surgical core variables and should include information on: **Use of energy-based devices for hemostasis** | 5.693 | 48 | 27 | No consensus | N/A | N/A | Expert opinion |
| 13 | Short-term postoperative follow-up data necessary for assessing prevalence of early postoperative hypoparathyroidism should include serum PTH levels on the day of surgery or POD1 (based on surgeon/institutional protocol). In the case of low serum PTH or symptoms of hypocalcemia, subsequent measurements of ionized or albumin-adjusted serum calcium are needed in the postoperative period (days-weeks) to assess any need for, or guide treatment of hypoparathyroidism | 7.370 | 10 | 9 | Near-consensus | N/A | Agree: 79.79%, Disagree: 20.21% | Moderate |
|  | Short-term postoperative follow-up data necessary for assessing prevalence of early postoperative hypoparathyroidism should include serum PTH levels on the day of surgery or POD1 (based on surgeon/institutional protocol). In the case of low serum PTH or symptoms of hypocalcemia **or unavailability of serum PTH measurement**, subsequent measurements of ionized or albumin-adjusted serum calcium **and phosphates** are needed in the postoperative period (days-weeks) to assess any need for, or guide treatment of hypoparathyroidism | 7.119 | 16 | 14 | N/A | Near-consensus | Agree: 60.14%, Disagree: 39.86% | Expert opinion |
| 14 | Short-term postoperative follow-up data should also include use of calcium supplementation (routine vs. selective; oral only vs. intravenous) and vitamin D or active vitamin D analogue prescription | 8.370 | 3 | 1 | Consensus | N/A | N/A | Moderate |
| 15 | To define prevalence of symptomatic hypoparathyroidism description of symptoms, and how symptoms/signs were assessed and documented (patient-reported symptoms vs. clinician-reported signs) should be specified. In particular, need for intravenous calcium, visit(s) to the emergency department, and readmission(s) for hypoparathyroidism should be reported | 8.179 | 5 | 1 | Consensus | N/A | N/A | Moderate |
| 16 | Long-term follow-up data necessary for assessing prevalence of permanent hypoparathyroidism should include serum calcium (albumin-adjusted and/or ionized) and PTH levels at 12 months after surgery, and information if an attempt was undertaken at stopping supplements within 12 months postoperatively with failure to stay off supplements due to low calcium and/or reoccurring symptoms | 8.146 | 8 | 4 | Consensus | N/A | N/A | Moderate |
| 17 | Pathology data should be reported with number (full/partial) of parathyroids identified in specimen | 7.876 | 6 | 3 | Consensus | N/A | N/A | Moderate |
| 18 | In addition, preoperative serum PTH levels (low vs. normal vs. high) and surgical experience (low-volume: ≤25 thyroid procedures per year vs. high-volume > 50 thyroid procedures per year) along with years of practice might be helpful optional parameters describing landscape of risk factors for postoperative hypoparathyroidism | 6.719 | 24 | 9 | Near-consensus | N/A | N/A | Moderate |
|  | In addition, preoperative serum PTH levels (low vs. normal vs. high) and surgical experience (low-volume: ≤25 thyroid procedures per year vs. high-volume > 50 thyroid procedures per year) along with years of practice might be helpful optional parameters describing landscape of risk factors for postoperative hypoparathyroidism | 7.142 | 14 | 6 | N/A | Consensus | N/A | Moderate |

^a^ For near consensus approved/rejected by the general assembly vote

^b^ GA: General assembly vote, applicable only for statements with near-consensus

N/A: not applicable

Supplementary Table S3: Summary of statements concerning quality markers and metrics needed to be reported in surgical research and publications concerning postoperative hypoparathyroidism following total or completion thyroidectomy.

| **Statement No** | **Statement text** | **Mean score**  **(n=92 experts)** | **Outliers**  **No** | **Negative outliers**  **No** | **Status after Delphi process part 1^a^** | **Status after Delphi process part 2^b^** | **GA vote^b^**  **Agree -Disagree**  **(n=297 experts, including 88 ESES, 111 AAES, and 98 IAES)** | **Quality of evidence** |
| --- | --- | --- | --- | --- | --- | --- | --- | --- |
| 19 | Quality markers and metrics of thyroid surgery related to postoperative hypoparathyroidism should be reported in research and publications to allow for benchmarking analysis and quality improvement. | 7.840 | 6 | 3 | Consensus |  |  | Moderate |
| 20 | The preferred way of reporting should be through the multicentre (e.g. NSQIP, EUROCRINE, CESQIP etc.) or national (e.g. SQRTPA, UKRETS etc.) databases that audit the workload and monitor outcome measures after thyroid surgery. However, the membership to these databases remains optional in the vast majority of health care environments. | 6.920 | 25 | 8 | Near-consensus | N/A | N/A | Low |
|  | The preferred way of reporting should be through the multicentre (e.g. NSQIP, EUROCRINE, CESQIP etc.) or national (e.g. SQRTPA, UKRETS etc.) databases that audit the workload and monitor outcome measures after thyroid surgery. However, the membership to these databases remains optional in the vast majority of health care environments. | 7.108 | 17 | 7 | N/A | Near-consensus | Agree: 82.87%, Disagree: 17.13% | Low |
| 21 | Serum PTH level (when possible) should be obtained within 24hours after total or completion thyroidectomy as an obligatory surrogate biochemical marker of postoperative hypoparathyroidism. This parameter impacts management at the time of discharge. In addition, PTH level may be used as quality marker allowing for estimation of the prevalence of early hypoparathyroidism, and also to exclude a high prevalence of permanent postoperative hypoparathyroidism. | 7.590 | 12 | 10 | Near-consensus | N/A | Agree: 69.75%, Disgree: 30.25% | High |
|  | Serum PTH level (when possible **and available**) should be obtained within **24 hours (preferably 1 to 6 hours)** after total or completion thyroidectomy as an obligatory surrogate biochemical marker of postoperative hypoparathyroidism. This parameter impacts management at the time of discharge. In addition, PTH level may be used as quality marker allowing for estimation of the prevalence of early hypoparathyroidism, and also to exclude a high prevalence of permanent postoperative hypoparathyroidism. | 7.74 | 10 | 9 | N/A | Near-consensus | Agree: 66.90%, Disagree: 33.10% | Moderate |
| 21A | Patients with normal PTH serum levels on POD1 have a very low risk of permanent hypoparathyroidism and are expected to need minimal monitoring and no calcium supplementation at discharge. | 7.863 | 6 | 2 | Consensus | N/A | N/A | High |
| 21B | Undetectable PTH serum level within 24 hours after thyroid surgery may increase the risk of permanent parathyroid dysfunction and these patients require immediate onset of supplementation with calcium and active vitamin D analogue and further close follow-up. | 7.965 | 6 | 4 | Consensus | N/A | N/A | Moderate |
| 22 | Some preoperative parameters, intraoperative events, and postoperative follow-up data are obligatory to be reported in publications as they are considered quality metrics of thyroid surgery which allow for more comprehensive risk assessment of permanent hypoparathyroidism state. | 7.704 | 9 | 2 | Consensus | N/A | N/A | Moderate |
| 22A | Some preoperative parameters like 25-OH vitamin D serum level, and PTH serum level should be reported. | 6.863 | 27 | 10 | Near-consensus | N/A | N/A | Moderate |
|  | Some preoperative parameters like 25-OH vitamin D serum level, and PTH serum level should be reported **when possible and available.** | 7.879 | 3 | 2 | N/A | Consensus | N/A | Moderate |
| 22B | Some intraoperative events like number of parathyroid glands identified visually with confidence / preserved in situ / reimplanted should be reported. | 7.613 | 7 | 5 | Consensus | N/A | N/A | Moderate |
| 22C | Some postoperative follow-up data including inadvertent excision of parathyroid gland(s) confirmed on histology, PTH serum level within 24 hours of the operation, need for intravenous calcium, visit(s) to the emergency department, and readmission(s) for hypoparathyroidism, use of calcium/vitD supplements during early (6 weeks) and long-term (12 months) follow-up should be reported. | 8.045 | 7 | 2 | Consensus | N/A | N/A | Moderate |
| 23 | The number of data points and the length of follow-up needed in quality registries to make a perfect calculation of transient and true permanent hypoparathyroidism rate after thyroid surgery may be difficult to reach. Hence, alternatively active vitamin D analogue use or prescription for over 12 months after thyroid surgery collected from a secondary source such as an insurance claims registry or a national prescribed drug register may be a feasible approximation, although it probably underestimates the true prevalence of permanent hypoparathyroidism somewhat. | 6.568 | 26 | 17 | Near-consensus | N/A | Agree: 36.60%, Disagree: 63.40% | Low |
|  | The number of data points and the length of follow-up needed in quality registries to make a perfect calculation of transient and true permanent hypoparathyroidism rate after thyroid surgery may be difficult to reach. Hence, alternatively active vitamin D analogue use or prescription for over 12 months after thyroid surgery collected from a secondary source such as an insurance claims registry or a national prescribed drug register may be a feasible approximation, although it probably underestimates **or overestimates** the true prevalence of permanent hypoparathyroidism somewhat. | 6.722 | 10 | 10 | N/A | Near-consensus | Agree: 72,32%, Disagree: 27.68% | Low |

^a^ For near consensus approved/rejected by the general assembly vote

^b^ GA: General assembly vote, applicable only for statements with near-consensus

N/A: not applicable
